# Comparative Histological Subtyping of Immune Cell Infiltrates in MPO-ANCA and PR3-ANCA Glomerulonephritis

**DOI:** 10.3389/fimmu.2021.737708

**Published:** 2021-10-21

**Authors:** Samy Hakroush, Désirée Tampe, Philipp Ströbel, Peter Korsten, Björn Tampe

**Affiliations:** ^1^ Institute of Pathology, University Medical Center Göttingen, Göttingen, Germany; ^2^ Department of Nephrology and Rheumatology, University Medical Center Göttingen, Göttingen, Germany

**Keywords:** immune cell infiltration, autoimmune diseases, systemic vasculitis, ANCA-associated glomerulonephritis, ANCA subtypes

## Abstract

**Background:**

Acute kidney injury (AKI) is a common and severe complication of anti-neutrophil cytoplasmic antibodies (ANCA)-associated vasculitis (AAV), potentially leading to chronic kidney disease (CKD), end-stage renal disease (ESRD), or death. Pathogenic ANCAs, in particular proteinase 3 (PR3) and myeloperoxidase (MPO), trigger a deleterious immune response with intrarenal immune cell infiltration resulting in a pauci-immune necrotizing and crescentic glomerulonephritis (GN). However, a systematic analysis of intrarenal immune cell subtypes concerning neutrophils, eosinophils, plasma cells, and mononuclear cell infiltrates (macrophages, lymphocytes) in ANCA GN remains elusive. Therefore, we aimed to compare distinct immune cell infiltrates in association with clinicopathological findings in ANCA GN.

**Methods:**

A total of 53 kidney biopsies with ANCA GN at the University Medical Center Göttingen were retrospectively analyzed. Histological infiltrates of neutrophils, eosinophils, plasma cells, and mononucleated cells (macrophages, lymphocytes) were quantified as a fraction of the total area of inflammation.

**Results:**

Neutrophilic infiltrates were associated with glomerular necrosis and severe kidney injury in ANCA GN. Among tubulointerstitial lesions, intrarenal neutrophils correlated with interstitial inflammation, tubulitis, and inflammation in areas of interstitial fibrosis/tubular atrophy (IFTA), representing active inflammatory lesions. Concerning eosinophils, infiltrates were associated with severe kidney injury, interstitial inflammation, and cellular casts independent of glomerular lesions, implicating a distinct role in inflammation and damage in ANCA GN. Plasma cell infiltrates correlated with tubulitis and interstitial fibrosis and were associated with renal replacement therapy during the short-term disease course. Finally, mononuclear cell infiltrates correlated with severe kidney injury and active histopathological lesions (glomerular crescents, interstitial inflammation, tubulitis, inflammation, and tubulitis in areas of IFTA) besides chronic lesions (interstitial fibrosis and tubular atrophy) in ANCA GN. Interestingly, intrarenal subtypes of immune cell infiltrates differed in MPO-ANCA versus PR3-ANCA GN and were associated with distinct glomerular and tubulointerstitial lesions, implicating different pathogenic mechanisms of kidney injury in ANCA subtypes.

**Conclusion:**

Our observations imply distinct pathomechanisms contributing to inflammation and renal injury in MPO vs. PR3-associated ANCA GN and potentially contribute to new therapeutic targets in specific ANCA subtypes.

## Introduction

According to the 2012 revised Chapel Hill Consensus Conference Nomenclature of Vasculitides, anti-neutrophil cytoplasmic antibody (ANCA)-associated vasculitis (AAV) is a small vessel vasculitis, most frequently presenting as microscopic polyangiitis (MPA) or granulomatosis with polyangiitis (GPA) (1, 2). Acute kidney injury (AKI) is a common and severe complication of AAV as it can cause progressive chronic kidney disease (CKD), end-stage renal disease (ESRD), or death (3, 4). Pathogenic ANCAs, in particular proteinase 3 (PR3) and myeloperoxidase (MPO), trigger a deleterious immune response resulting in a pauci-immune necrotizing and crescentic glomerulonephritis (GN), a common manifestation of glomerular injury in AAV (5). Several studies have investigated determinants of renal outcomes in ANCA GN, including baseline kidney function, acute and chronic histopathological lesions (6–9). On a mechanistic level, neutrophils are activated by pathogenic ANCAs, causing a release of inflammatory cytokines, reactive oxygen species, and lytic enzymes, resulting in an excessive formation of neutrophil extracellular traps (NETs) (10–12). Besides neutrophils, plasma cell infiltration is also commonly observed in ANCA GN and is associated with active and early disease (13). While neutrophil and plasma cell infiltrations predominate the early phase of inflammation in AAV, the neutrophil-rich necrotizing lesions are converted into a monocyte/macrophage-rich granuloma. This is accompanied by an infiltration of lymphocytes, such as T cells (14, 15). We previously established that MPO-ANCA GN more frequently shows active tubulointerstitial lesions, including total inflammation (16). However, a systematic analysis of intrarenal immune cell subtypes with regard to neutrophils, eosinophils, plasma cells, and mononuclear cell infiltrates (macrophages, lymphocytes) in ANCA GN remains elusive. Therefore, we aimed to compare distinct immune cell infiltrates in association with clinicopathological findings in a cohort of patients with biopsy-proven ANCA GN.

## Methods

### Study Population

A total number of 53 kidney biopsies with ANCA GN at the University Medical Center Göttingen were retrospectively included between 2015 till 2020; the patient cohort has previously been described (16–21). While no formal approval was required for the use of routine clinical data, a favorable ethical opinion was granted by the local Ethics committee (no. 22/2/14 and 28/09/17). At admission, the Birmingham Vasculitis Activity Score (BVAS) version 3 was assessed (22). Medical records were used to obtain data on age, sex, duration of disease onset before admission, diagnosis (MPA or GPA), and laboratory results. The estimated glomerular filtration rate (eGFR) was calculated using the Chronic Kidney Disease Epidemiology Collaboration (CKD-EPI) equation (23). When required, renal replacement therapy (RRT) was performed intermittently in all cases. Indications for RRT included severe electrolyte and acid-base abnormalities, volume overload, or encephalopathy. Glucocorticoids (GCs) were administered either as intravenous pulse therapy or orally with a tapering schedule. At time of kidney biopsy, all patients received GCs and further remission induction therapy was initiated thereafter based on histopathological confirmation of ANCA GN.

### Renal Histopathology

Two pathologists (SH and PS) evaluated the kidney biopsies and were blinded to clinical data. Within a kidney biopsy, infiltrates of neutrophils, eosinophils, plasma cells, and mononucleated cells (macrophages, lymphocytes) were quantified as a fraction of the area of total cortical inflammation. The total cortical inflammation including areas of interstitial fibrosis and tubular atrophy, subcapsular and perivascular cortex including nodular infiltrates were considered. In addition, each glomerulus was scored separately for the presence of necrosis, crescents, and global sclerosis. Based on these scorings, histopathological subgrouping according to Berden et al. into focal, crescentic, mixed, or sclerotic classes was performed (6). Furthermore, the ANCA renal risk score (ARRS), according to Brix et al. into low, medium, or high risk, was calculated (7). The total renal chronicity score including global/segmental glomerular sclerosis (score 0: <10%, 1: 10-25%, 2: 26-50%, 3: >50%), interstitial fibrosis (score 0: <10%, 1: 10-25%, 2: 26-50%, 3: >50%), tubular atrophy (score 0: <10%, 1: 10-25%, 2: 26-50%, 3: >50%), and arteriosclerosis (score 0: intimal thickening<thickness of media, 1: intimal thickening≥thickness of media) was evaluated as previously described (24). Kidney biopsies were also evaluated analogously to the Banff scoring system for allograft pathology, as described previously (25). In brief, Banff score lesions include interstitial inflammation (*i*), tubulitis (*t*), arteritis (*v*), glomerulitis (*g*), interstitial fibrosis (*ci*), tubular atrophy (*ct*), arteriolar hyalinosis (*ah*), peritubular capillaritis (*ptc*), total inflammation (*ti*), inflammation in areas of IFTA (*i-IFTA*) and tubulitis in areas of IFTA (*t-IFTA*) (25). Systematic histological scoring of tubular injury lesions was evaluated as previously described (26, 27). In brief, epithelial simplification and tubular dilation, non-isometric cell vacuolization, cellular, red blood cell (RBC), and hyaline casts were given a score ranging from 0 to 4 as a percentage of the total affected cortical area of the biopsy (score 0: <1%, 1: ≥1-10%, 2: ≥10-25%, 3: ≥25-50%, 4: >50%).

### Statistical Methods

Variables were tested for normal distribution using the Shapiro-Wilk test. Statistical comparisons were not formally powered or prespecified. Non-normally distributed continuous variables are shown as the median and interquartile range (IQR), categorical variables are presented as frequency and percentage. Spearman’s correlation was performed to assess the correlation between clinical, laboratory, and histopathological parameters, and heatmaps reflecting the mean values of Spearman’s ρ are shown, the asterisks indicating statistical significance correlations. Data analyses were performed with GraphPad Prism (version 8.4.3 for macOS, GraphPad Software, San Diego, California, USA). Multiple regression analyses were performed using IBM SPSS Statistics (version 27 for MacOS, IBM Corporation, Armonk, NY, USA). A probability (*p*) value of <*0.05* was considered statistically significant.

## Results

### Description of Demographic and Clinical Characteristics

**Figure 1:**
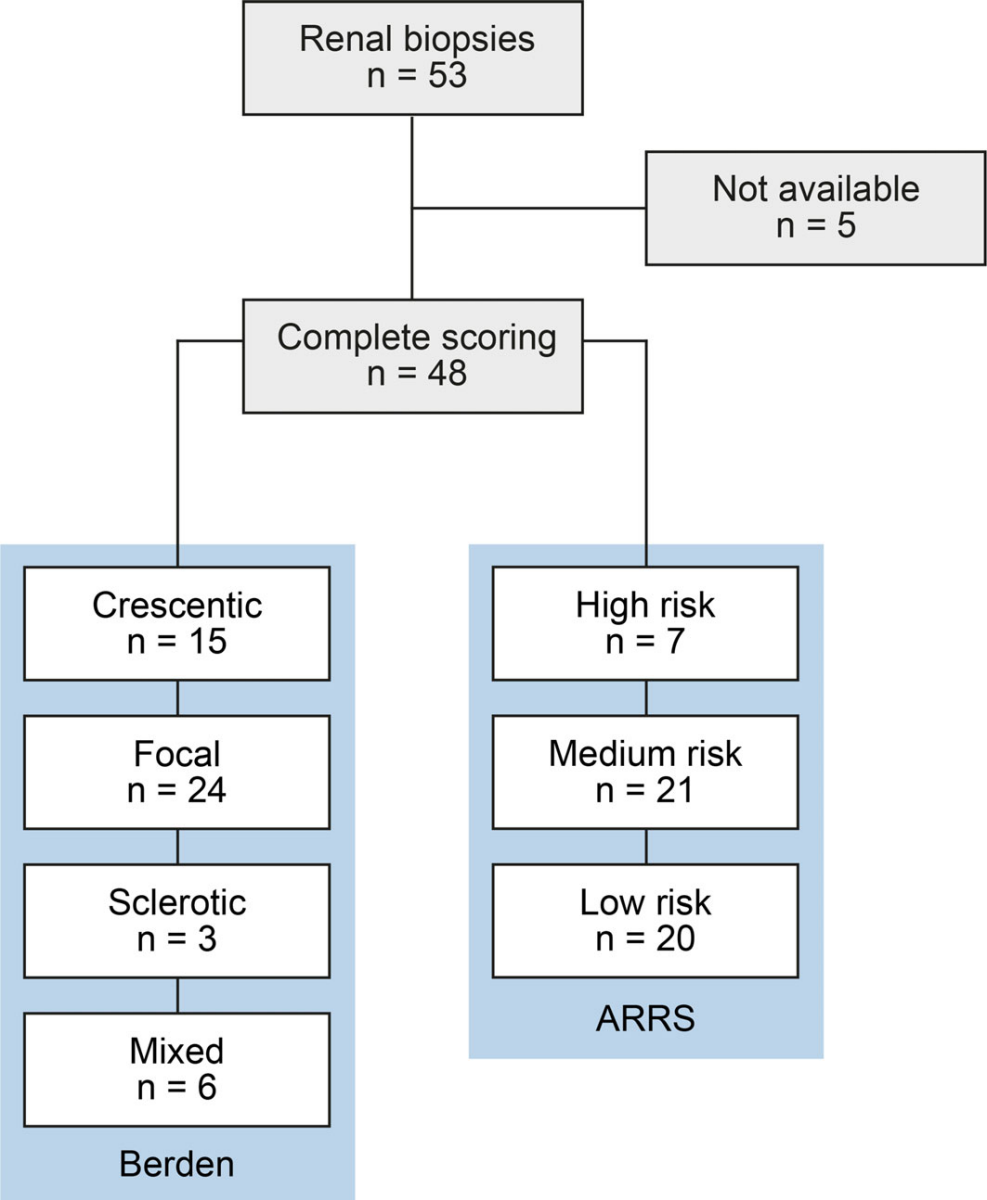
Total patient cohort of ANCA GN. STROBE flow chart of patient disposition with systematic analysis of distinct subtypes of intrarenal immune cell infiltrates in ANCA GN. ANCA, anti-neutrophil cytoplasmic antibodies; GN, glomerulonephritis; STROBE, Strengthening the Reporting of Observational Studies in Epidemiology.

**Figure 2:**
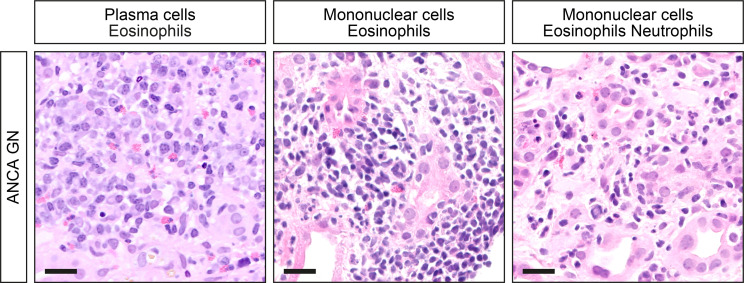
Distinct subtypes of intrarenal immune cell infiltrates in ANCA GN. Representative photomicrographs with inflammatory infiltrates are shown (scale bars: 20 μm). ANCA, anti-neutrophil cytoplasmic antibodies; GN, glomerulonephritis.

**Figure 3:**
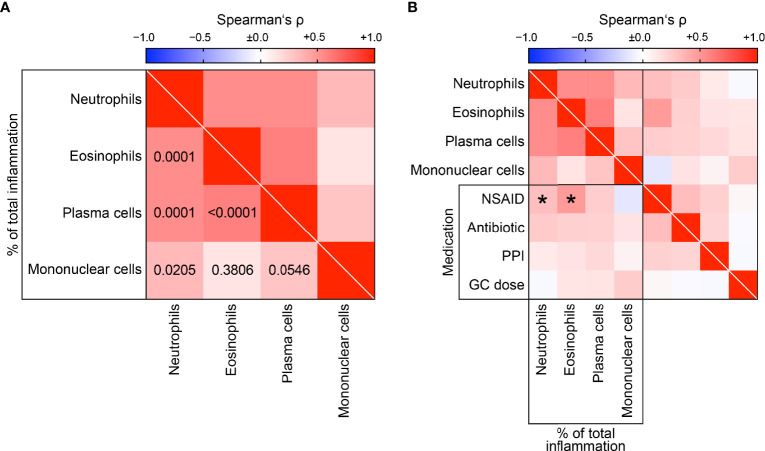
Correlative analysis of distinct intrarenal immune cell infiltrates. **(A)** Association between distinct subtypes of immune cell infiltrates in ANCA GN are shown by heatmap reflecting mean values of Spearman’s ρ, respective p values are shown. **(B)** Distinct subtypes of immune cell infiltrates in association with medication are shown by heatmap reflecting mean values of Spearman’s ρ, asterisks indicate *p < 0.05*. ANCA, anti-neutrophil cytoplasmic antibodies; GC, glucocorticoid; GN, glomerulonephritis; NSAID, non-steroidal anti-inflammatory drug; PPI, proton-pump inhibitor.

A total number of 53 kidney biopsies with ANCA GN were retrospectively included from 2015 to 2021. Complete scoring of immune cell infiltrates was obtained in 48 kidney biopsies with confirmed ANCA GN (**Figure 1**), including 2 patients with a consecutive kidney biopsy due to relapse of ANCA GN. Histopathological subgrouping revealed 15/48 (31.3%) crescentic, 24/48 (50%) focal, 3/48 (6.3%) sclerotic and 6/48 (12.5%) mixed class ANCA GN (6). ARRS was high in 7/48 (14.6%), intermediate in 21/48 (43.8%), and low-risk class ANCA GN in 20/48 (41.7%) of cases (**Figure 1**) (7). The baseline characteristics of the cohort are shown in **Table 1**. In this cohort, the median age at diagnosis was 64 (54.25-73.75) years. All patients were Caucasian, and 20/48 (41.7%) were female. The median disease onset before admission was 17.5 (7-40) days; a kidney biopsy was performed within 6 (3-9) days after admission to confirm renal involvement of AAV. Based on clinical characteristics, 24/48 (50%) patients were diagnosed as MPA and the remainder as GPA. A total number of 7/48 (14.6%) patients had a history of vasculitis; the median (IQR) BVAS was 18 (15-20). There were 14/48 patients (83.3%) with extrarenal manifestations of AAV (27 with lung, 7 with sinus, 12 with joint, 3 with ear, 3 with eye, 6 with peripheral nerve, and 9 with skin involvement), and 7/48 (14.6%) had an alveolar hemorrhage. Based on laboratory findings, 23/48 (47.9%) had positive myeloperoxidase (MPO) and 25/48 (52.1%) positive proteinase 3 (PR3) ANCA. The worst median eGFR at disease onset was 18.3 (9.65-50.85) mL/min/1.73 m^2^, and 15/48 (31.3%) required RRT within 30 days after admission. Systematic histological scoring of intrarenal immune cell subtypes in ANCA GN revealed that a neutrophilic infiltrate was detectable in 16/48 (33.3%), an eosinophilic infiltrate in 10/48 (20.8%), a plasma cell infiltrate in 32/48 (66.7%), and a mononuclear (macrophages, lymphocytes) cell infiltrate in 43/48 (89.6%, **Figure 2** and **Table 2**) of evaluable biopsies. To elucidate the association between infiltrates of immune cell subtypes, we next quantified the presence of neutrophils, eosinophils, plasma cells, and mononucleated cells as a fraction of the total area. There was a strong correlation of immune cell subtypes with each other; in particular, the presence of neutrophils correlated with eosinophils, plasma cells and mononuclear cells, and eosinophils with plasma cells (**Figure 3A**), respectively. Because medication with a non-steroidal anti-inflammatory drug (NSAID), antibiotics, or proton-pump inhibitors (PPI) potentially triggers an interstitial inflammation in the kidney, we next analyzed a correlation between distinct intrarenal immune cell subtypes and these medications in addition to GC dose at time of kidney biopsy. We observed an association between NSAID use and neutrophilic (r=0.3074, *p=0.0336*) as well as eosinophilic cell infiltrates (r=0.4682, *p=0.0008*, **Figure 3B**). In summary, subtypes of immune cells in kidney biopsies were observed in a majority of ANCA GN cases. In addition, we observed an association between distinct immune cell infiltrates and prior NSAID use.

**Table 1:** Clinical and laboratory parameters of the total ANCA GN cohort.

Parameter	Value
*Clinical data*	
Median age (IQR) – years	64 (54.25-73.75)
Female sex – no. (%)	20 (41.7)
Disease onset – days before admission (IQR)	17.5 (7-40)
Kidney biopsy – days after admission (IQR)	6 (3-9)
ANCA subtype MPA/GPA – no. (%)	24/24 (50/50)
History of vasculitis – no. (%)	7 (14.6)
RRT within 30 days after admission – no. (%)	15 (31.3)
*AAV manifestations*	
Median BVAS (IQR) – points	18 (15-20)
Extrarenal manifestation – no. (%)	40 (83.3)
Lung involvement – no. (%)	27 (56.3)
Pulmonary hemorrhage – no. (%)	7 (14.6)
Sinus involvement – no. (%)	7 (14.6)
Joint involvement – no. (%)	12 (25)
Ear involvement – no. (%)	3 (6.3)
Eye involvement – no. (%)	3 (6.3)
Nerve involvement – no. (%)	6 (12.5)
Skin involvement – no. (%)	9 (18.8)
*Laboratory data*	
ANCA subtype MPO/PR3 – no. (%)	23 (47.9)
Median serum creatinine (IQR) – mg/dL	3.06 (1.355-5.138)
Median eGFR (IQR) – mL/min/1.73 m^2^	18.3 (9.65-50.85)
Median CRP (IQR) – mg/L	60.5 (20.05-109.5)
*Urinary data*	
Median uPCR (IQR) – mg/g	940.5 (523.6-1559)
Median uACR (IQR) – mg/g	451.4 (156.3-855.3)
Median α_1_-microglobulin (IQR) – mg/g	65.9 (34.8-178.2)
Median α_2_-macroglobulin (IQR) – mg/g	4.98 (2.903-10.3)
Median IgG (IQR) – mg/g	44.33 (21.63-201.5)
Acanthocytes – no. (%)	6 (12.5)
*Histopathological subgrouping*	
Crescentic class – no. (%)	15 (31.3)
Focal class – no. (%)	24 (50)
Sclerotic class – no. (%)	3 (6.3)
Mixed class – no. (%)	6 (12.5)
*ARRS*	
High risk – no. (%)	7 (14.6)
Medium risk – no. (%)	21 (43.8)
Low risk – no. (%)	20 (41.7)
*Total renal chronicity*	
Score (IQR) – points	3 (1.25-5)

ANCA, anti-neutrophil cytoplasmic antibodies; ARRS, ANCA renal risk score; BVAS, Birmingham Vasculitis Activity Score; CRP, C-reactive protein; eGFR, estimated glomerular filtration rate (CKD-EPI); GN, glomerulonephritis; GPA, granulomatosis with polyangiitis; IQR, interquartile range; MPA, microscopic polyangiitis; MPO, myeloperoxidase; no., number; PR3, proteinase 3; RRT, renal replacement therapy; uPCR, urinary protein-to-creatinine ratio; uACR, urinary albumin-to-creatinine ratio.

**Table 2:** Systematic histological scoring of intrarenal immune cell subtypes in ANCA GN.

Immune cell subtype	Value
Neutrophils – no. (%) Eosinophils – no. (%) Plasma cells – no. (%) Mononucleated cells – no. (%)	16/48 (33.3) 10/48 (20.8) 32/48 (66.7) 43/48 (89.6)

ANCA, anti-neutrophil cytoplasmic antibodies; GN, glomerulonephritis; no., number.

### Distinct Clinical Parameters and Laboratory Markers in Association With Intrarenal Immune Cell Subtypes

**Figure 4:**
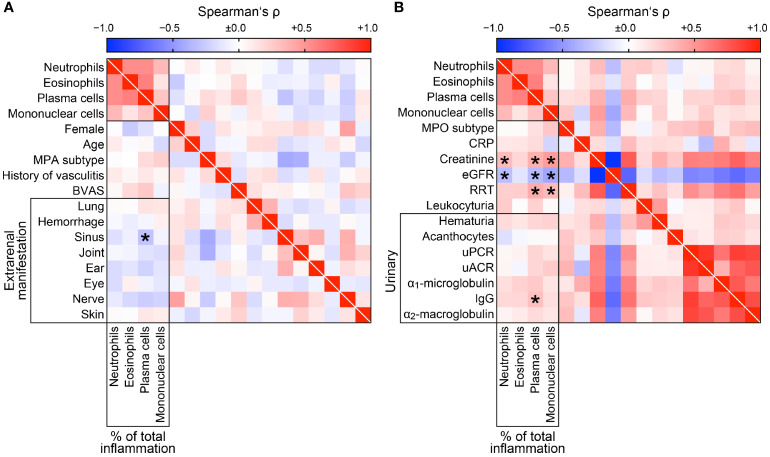
Distinct clinical parameters and laboratory markers in association with intrarenal immune cell subtypes. **(A)** Distinct subtypes of immune cell infiltrates in association with clinical characteristics at disease manifestation of AAV are shown by heatmap reflecting mean values of Spearman’s ρ, asterisks indicate *p < 0.05*. **(B)** Distinct subtypes of immune cell infiltrates in association with laboratory parameters at disease manifestation of AAV are shown by heatmap reflecting mean values of Spearman’s ρ, asterisks indicate *p < 0.05*. AAV, ANCA-associated vasculitis; ANCA, anti-neutrophil cytoplasmic antibodies; BVAS, Birmingham Vasculitis Activity Score; CRP, C-reactive protein; eGFR, estimated glomerular filtration rate; IgG, immunoglobulin G; MPA, microscopic polyangiitis; MPO, myeloperoxidase; RRT, renal replacement therapy; uACR, urinary albumin-to-creatinine ratio; uPCR, urinary protein-to-creatinine ratio.

We next analyzed intrarenal subtypes of immune cell infiltrates in association with clinical parameters, laboratory, and urinary markers in AAV. The only association with clinical parameters was observed between plasma cell infiltrates and less frequent sinus involvement in AAV (r=-0.2954, *p=0.0415*, **Figure 4A**). Neutrophilic, plasma cellular, and mononuclear infiltrates correlated with plasma creatinine (r=0.3217, *p=0.0258*, r=0.3745, *p=0.0087*, r=0.4608, *p=0.0010*, respectively) and eGFR loss (r=-0.3141, *p=0.0297*, r=-0.3330, *p=0.0207*, r=-0.4278, *p=0.0024*, **Figure 4B**). Interestingly, intrarenal infiltrates of plasma (r=0.3936, *p=0.0056*) and mononuclear cells (r=0.3345, *p=0.0201*) were also associated with severe kidney injury requiring RRT during the initial course of the disease (**Figure 4B**). Multiple regression analysis comparing all immune cell infiltrates confirmed that presence of mononuclear cells was associated with severe kidney injury reflected by RRT requirement during the initial disease course (**Table 3**), confirming an important of mononuclear cell infiltrates in the early phase of ANCA GN. In contrast, intrarenal immune cell subtypes did not correlate with urinary findings despite an association between plasma cellular infiltrates and urinary IgG levels (**Figure 4B**). Furthermore, intrarenal eosinophils did not correlate with any clinical parameter, laboratory, or urinary marker in AAV. In summary, systematic scoring of intrarenal immune cell subtypes revealed that distinct immune cell types were specifically associated with a deterioration of kidney function independent of clinical parameters, laboratory or urinary markers in AAV.

**Table 3:** Multiple regression analyses of parameters associated with RRT within 30 days after admission.

Immune cell subtype	ß	SE	p value
Neutrophils – no. (%) Eosinophils – no. (%) Plasma cells – no. (%) Mononucleated cells – no. (%)	0.0913 0.2002 0.0884 0.3263	4.7108 6.6283 1.6159 0.7044	*0.5621* *0.2967* *0.6341* *0.0277*

RRT, renal replacement therapy; SE, standard error.

### Intrarenal Subtypes of Immune Cell Infiltrates Correlate With Active ANCA GN

**Figure 5:**
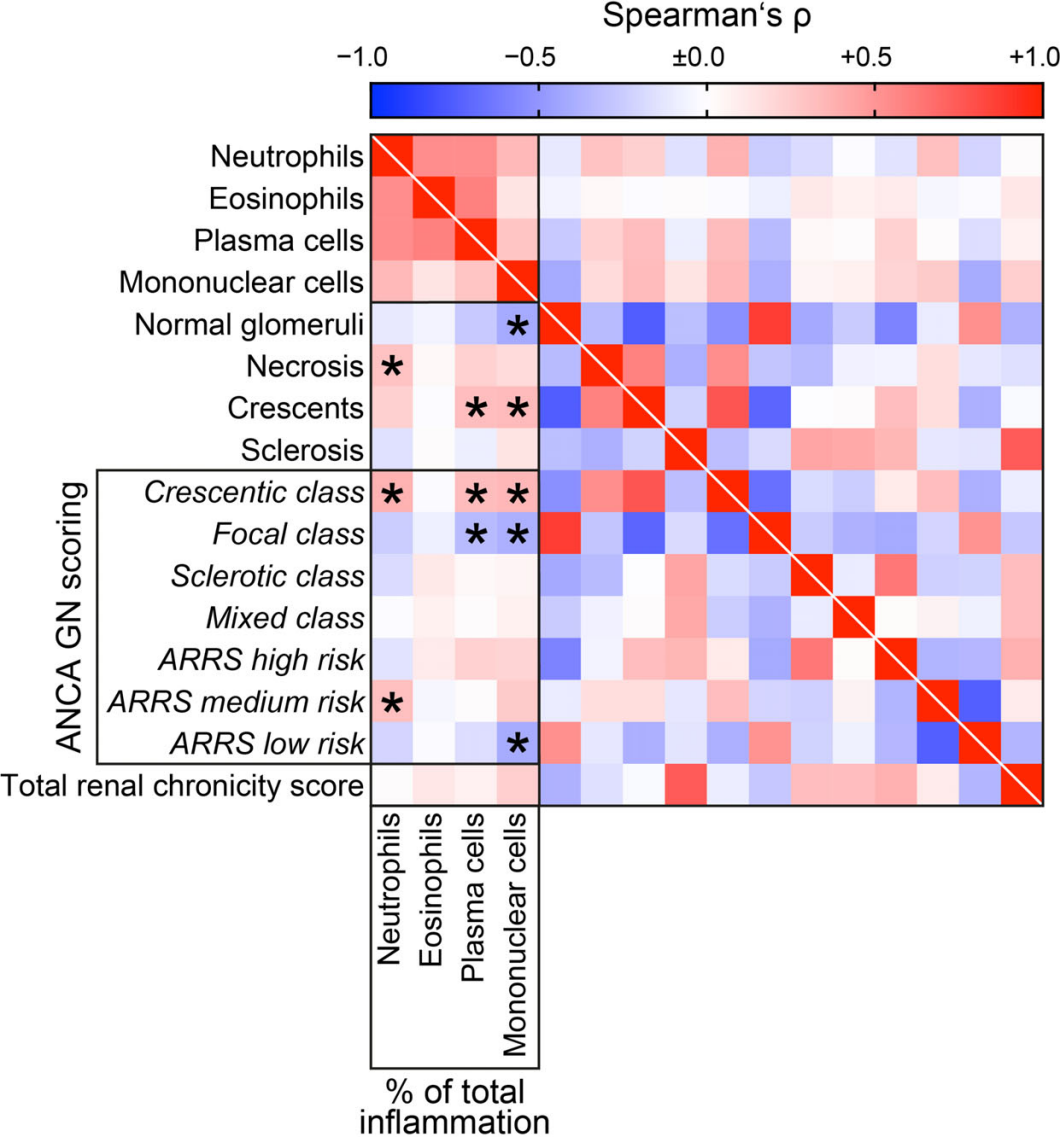
Intrarenal subtypes of immune cell infiltrates correlate with active ANCA GN. Distinct subtypes of immune cell infiltrates in association with established histopathological scoring of ANCA GN are shown by heatmap reflecting mean values of Spearman’s ρ, asterisks indicate *p < 0.05*. ANCA, anti-neutrophil cytoplasmic antibodies; GN, glomerulonephritis.

Since pauci-immune necrotizing and crescentic GN is a common manifestation of glomerular injury in AAV, we directly correlated intrarenal immune cell subtypes with established histopathological scores of ANCA GN (6–8). Neutrophilic (r=0.3675, *p=0.0102*), plasma cell (r=0.3225, *p=0.0254*), and mononuclear infiltrates (r=0.3393, *p=0.0183*) correlated with crescentic class ANCA GN (**Figure 5**). Interestingly, infiltrates of plasma (r=0.3177, *p=0.0278*) and mononuclear cells (r=0.3319, *p=0.0212*) were associated with glomerular crescents, whereas neutrophilic infiltrates specifically correlated with glomerular necrosis (r=0.2864, *p=0.0484*, **Figure 5**). Again, intrarenal eosinophils did not correlate with any histopathological scoring of ANCA GN (**Figure 5**). Finally, there was no correlation between any immune cell infiltrate and total renal chronicity score in ANCA GN (**Figure 5**). In summary, systematic scoring of intrarenal immune cell subtypes revealed that distinct immune cell infiltrates were specifically associated with active ANCA GN, including glomerular crescents and necrosis.

### Intrarenal Subtypes of Immune Cell Infiltrates in Association With Tubulointerstitial Lesions in ANCA GN

**Figure 6:**
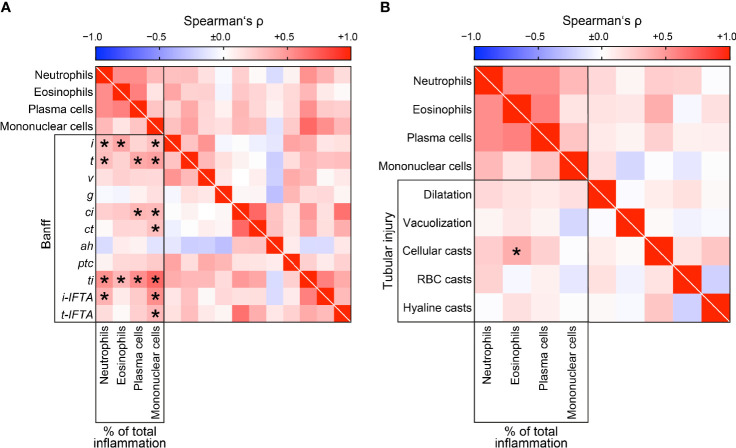
Intrarenal subtypes of immune cell infiltrates in association with tubulointerstitial lesions in ANCA GN. **(A)** Distinct subtypes of immune cell infiltrates in association with tubulointerstitial lesions analogous to the Banff scoring system are shown by heatmap reflecting mean values of Spearman’s ρ, asterisks indicate *p < 0.05*. **(B)** Distinct subtypes of immune cell infiltrates in association with ATI lesions are shown by heatmap reflecting mean values of Spearman’s ρ, asterisks indicate *p < 0.05*. *ah*, arteriolar hyalinosis; ANCA, anti-neutrophil cytoplasmic antibodies; ATI, acute tubular injury; *ci*, interstitial fibrosis; *ct*, tubular atrophy; *g*, glomerulitis; GN, glomerulonephritis; *i*, interstitial inflammation; *i-IFTA*, inflammation in IFTA; RBC, red blood cell; *t*, tubulitis; *ptc*, peritubular capillaritis; *ti*, total inflammation; *t-IFTA*, tubulitis in IFTA; *v*, intimal arteritis.

Next, we compared intrarenal immune cell subtypes with tubulointerstitial lesions in ANCA GN (25). Neutrophilic infiltrates were associated with interstitial inflammation (*i*, r=0.3010, *p=0.0420*), tubulitis (*t*, r=0.3090, *p=0.0366*), and inflammation in areas of IFTA (*i-IFTA*, r=0.3664, *p=0.0123*, **Figure 6A**). Eosinophils only correlated with interstitial inflammation (*i*, r=0.4245, *p=0.0033*, **Figure 6A**). Plasma cell infiltration correlated with tubulitis (*t*, r=0.4108, *p=0.0046*) and interstitial fibrosis (*ci*, r=0.3457, *p=0.0161*, **Figure 6A**). Finally, infiltrates of mononuclear cells correlated with interstitial inflammation (*i*, r=0.3034, *p=0.0404*), tubulitis (*t*, r=0.4459, *p=0.0019*), interstitial fibrosis (*ci*, r=0.3349, *p=0.0200*), tubular atrophy (*ct*, r=0.2997, *p=0.0430*), inflammation in areas of IFTA (*i-IFTA*, r=0.5244, *p=0.0002*), and tubulitis in areas of IFTA (*t-IFTA*, r=0.3392, *p=0.0211*, **Figure 6A**), reflecting active and chronic tubulointerstitial lesions. Among tubular injury lesions, eosinophilic infiltrates specifically correlated with cellular casts in ANCA GN (r=0.3836, *p=0.0085*, **Figure 6B**) (26). As expected, all infiltrates of inflammatory cell subtypes correlated with total inflammation (*ti*). In summary, intrarenal subtypes of immune cell infiltrates correlated with distinct active and chronic tubulointerstitial lesions in ANCA GN.

### Intrarenal Subtypes of Immune Cell Infiltrates in MPO-ANCA Versus PR3-ANCA GN

**Figure 7:**
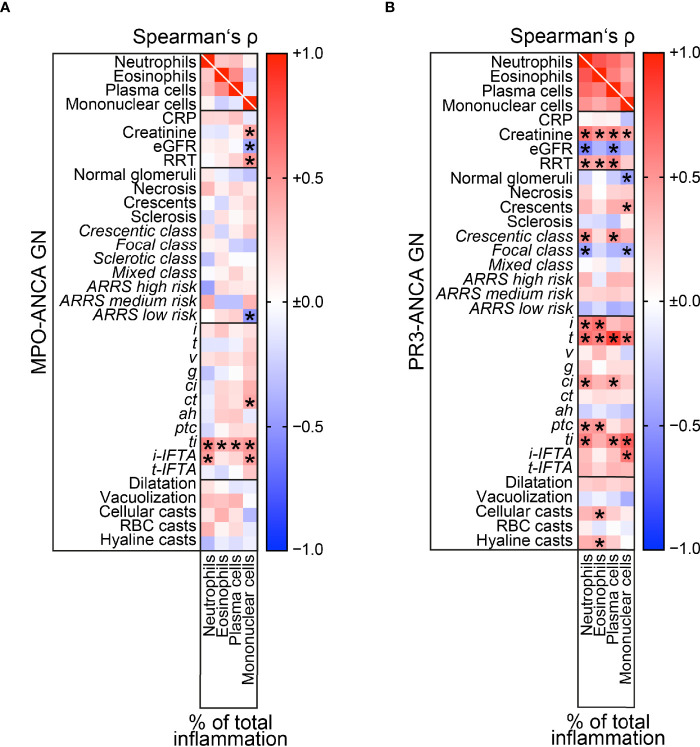
Intrarenal subtypes of immune cell infiltrates in MPO-ANCA versus PR3-ANCA GN. **(A)** Associations of clinical, laboratory parameters, and histopathological findings in MPO-ANCA GN are shown by heatmap reflecting mean values of Spearman’s ρ, asterisks indicate *p < 0.05*. **(B)** Associations of clinical, laboratory parameters and histopathological findings in PR3-ANCA GN are shown by heatmap reflecting mean values of Spearman’s ρ, asterisks indicate *p < 0.05*. *ah*, arteriolar hyalinosis; ANCA, anti-neutrophil cytoplasmic antibodies; ATI, acute tubular injury; BVAS, Birmingham Vasculitis Activity Score; *ci*, interstitial fibrosis; CRP, C-reactive protein; *ct*, tubular atrophy; eGFR, estimated glomerular filtration rate; *g*, glomerulitis; GN, glomerulonephritis; *i*, interstitial inflammation; IgG, immunoglobulin G; *i-IFTA*, inflammation in IFTA; MPA, microscopic polyangiitis; MPO, myeloperoxidase; RBC, red blood cell; RRT, renal replacement therapy; *t*, tubulitis; *ptc*, peritubular capillaritis; *ti*, total inflammation; *t-IFTA*, tubulitis in IFTA; uACR, urinary albumin-to-creatinine ratio; uPCR, urinary protein-to-creatinine ratio; *v*, intimal arteritis.

Finally, we analyzed intrarenal subtypes of immune cell infiltrates separately in MPO-ANCA versus PR3-ANCA GN. In MPO-ANCA GN, we observed a specific association between neutrophils (r=0.4576, *p=0.0281*) and mononuclear cells (r=0.4508, *p=0.0309*) with inflammation in areas of IFTA (*i-IFTA*, **Figure 7A**). Interestingly, a strong correlation between mononuclear cell infiltrates and severity of kidney injury reflected by rise of serum creatinine (r=0.4166, *p=0.0480*), eGFR loss (r=-0.4191, *p=0.0465*), and requirement of RRT (r=0.4893, *p=0.0178*), less categorization into ARRS low-risk group (r=-0.5083, *p=0.0133*), and accelerated tubular atrophy (*ct*, r=0.4172, *p=0.0477*) in MPO-ANCA GN was observed (**Figure 7A**). Again, all infiltrates of inflammatory cell subtypes correlated with total inflammation (*ti*) in MPO-ANCA GN (**Figure 7A**). In contrast to MPO-ANCA GN, the strongest association between neutrophilic infiltrates and severe kidney injury reflected by rise of serum creatinine (r=0.6281, *p=0.0008*), eGFR loss (r=-0.5805, *p=0.0023*), and requirement of RRT (r=0.4369, *p=0.0290*) was observed in PR3-ANCA GN (**Figure 7B**). Neutrophils in PR3-ANCA GN were correlated with crescentic class (r=0.5085, *p=0.0094*) and less focal class ANCA GN (r=-0.4525, *p=0.0232*), interstitial inflammation (*i*, r=0.5723, *p=0.0043*), tubulitis (*t*, r=0.6593, *p=0.0006*), interstitial fibrosis (*ci*, r=0.5022, *p=0.0105*), and peritubular capillaritis (*ptc*, r=0.4578, *p=0.0280*), reflecting active lesions in the majority of histopathological findings (**Figure 7B**). Interestingly, eosinophilic infiltrates were also associated with severe kidney injury independent of glomerular but active tubulointerstitial lesions reflected by interstitial inflammation (*i*, r=0.6688, *p=0.0005*), tubulitis (*t*, r=0.5780, *p=0.0039*), peritubular capillaritis (*ptc*, r=0.4863, *p=0.0186*), cellular (r=0.4347, *p=0.0382*) and hyaline casts (r=0.4184, *p=0.0469*, **Figure 7B**). In addition, plasma cell infiltrates were also associated with severe kidney injury reflected by rise of serum creatinine (r=0.5566, *p=0.0039*), eGFR loss (r=-0.5244, *p=0.0071*), and requirement of RRT (r=0.5361, *p=0.0057*), crescentic class ANCA GN (r=0.4957, *p=0.0117*), tubulitis (*t*, r=0.8622, *p<0.0001*), and interstitial fibrosis (*ci*, r=0.4393, *p=0.0280*, **Figure 7B**). Finally, intrarenal mononuclear cells correlated with decreased normal glomeruli (r=-0.4605, *p=0.0205*) and glomerular crescents (r=0.4184, *p=0.0374*), tubulitis (*t*, r=0.5926, *p=0.0029*), and inflammation in areas of IFTA (*i-IFTA*, r=0.6290, *p=0.0013*, **Figure 7B**). In summary, intrarenal subtypes of immune cell infiltrates were associated with distinct glomerular and tubulointerstitial lesions differing in MPO-ANCA versus PR3-ANCA GN, implicating that distinct signatures of immune cell infiltrates contribute to kidney injury in different ANCA subtypes.

## Discussion

We have previously shown that MPO-ANCA GN is associated with more severe deterioration of kidney function independent of systemic markers of AAV disease activity, associated with more interstitial vasculitis and total inflammation, as well as interstitial fibrosis and tubular atrophy (16). In addition, we previously observed tubulointerstitial inflammation was associated with presence of Bowman’s capsule ruptures, independently described to correlate with poor outcome in ANCA GN (20, 28, 29). Based on these previous observations that interstitial inflammation is frequently observed in the majority of ANCA GN cases, we here aimed to provide a comprehensive analysis of histological subtyping of immune cell infiltrates in association with clinicopathological findings in ANCA GN. At disease onset, neutrophils are activated by pathogenic ANCAs, causing a release of inflammatory cytokines, reactive oxygen species, and lytic enzymes, thus resulting in an excessive formation of neutrophil extracellular traps (NETs) (10–12). Besides neutrophils, plasma cell infiltration is also commonly observed in ANCA GN and has been associated with active and early disease (13). Intrarenal infiltrates of primed neutrophils are activated by MPO-ANCA and PR3-ANCA, leading to degranulation and the release of cytoplasmic granules into the glomerular and interstitial space in ANCA GN. As a result, reactive oxygen radicals (RORs) accumulate and cause vascular damage (11). In addition, stimulation of neutrophils causes the release of factors that activate the alternative complement system, aggravating vascular damage (30). Small vessel leakage of serum proteins and the formation of fibrin gives rise to fibrinoid necrosis (31). While neutrophil and plasma cell infiltrates predominate the early phase of inflammation in AAV, the neutrophil-rich necrotizing lesions are converted into a monocyte/macrophage-rich granuloma, and this is accompanied by infiltration of lymphocytes, such as T cells (14, 15).

This concept is in line with our observation that neutrophilic infiltrates were associated with glomerular necrosis and severe kidney injury in ANCA GN. Among tubulointerstitial lesions, intrarenal neutrophils correlated with interstitial inflammation, tubulitis, and inflammation in areas of IFTA representing active inflammatory lesions, again confirming the concept that activated neutrophils mediate inflammation and injury in the early phase of ANCA GN. Eosinophils do not contain MPO or PR3 but can be activated by ANCA-activated neutrophils. With regard to eosinophils, infiltrates were associated with cellular casts independent of glomerular lesions, implicating a distinct role in inflammation and tubular injury in ANCA GN. The pathogenic role of plasma cells in ANCA GN remains unclear, but local antibody production in the kidney may also contribute to interstitial inflammation in ANCA GN. Based on previous reports, B cell autoimmune response in AAV is facilitated by impaired T and B cell regulation and by B cell-stimulating factors released by activated neutrophils (32). Plasma cell-rich ANCA GN is frequently observed and has previously been associated with tubulointerstitial inflammation, implicating that plasma cell infiltrates may contribute to tubulitis (13, 33). This is confirmed by our observation that plasma cell infiltrates correlated with tubulitis and interstitial fibrosis. Finally, mononuclear cell infiltrates, including macrophages and lymphocytes, such as T cells, are found in ANCA GN (14, 15). Macrophages are a predominant immune cell subtype in ANCA GN infiltrating normal glomeruli and present in developing glomerular lesions (34). The presence of macrophages suggests an important role in renal injury and the recruitment of T cells, which dominate later stages in ANCA GN (35). Previous studies have shown that monocytes in ANCA GN localize to sites of active glomerular lesions, including fibrinoid necrosis, cellular crescents, and periglomerular inflammation (36). In addition, intrarenal macrophages display positivity for MPO and produce extracellular traps containing MPO, further contributing to MPO antigen presentation and aggravating renal injury (37). This concept concurs with our observation that mononuclear cell infiltrates not only correlate with chronic lesions, including interstitial fibrosis and tubular atrophy (suggesting a role in clearing injured tissue), but also correlated with active histopathological lesions including glomerular crescents, interstitial inflammation, and tubulitis (thereby probably contributing to active tubulointerstitial damage). Furthermore, presence of mononuclear cells was associated with severe kidney injury and RRT requirement during the initial disease course in multivariate comparison of immune cell infiltrates, confirming an important role also in the early phase of ANCA GN. Interestingly, intrarenal subtypes of immune cell infiltrates were associated with distinct glomerular and tubulointerstitial lesions and differed in MPO-ANCA versus PR3-ANCA GN, implicating that distinct signatures of immune cell infiltrates contribute to kidney injury in these two subtypes of the disease. The only association between medication and immune cell infiltration was observed for prior NSAID use and intrarenal eosinophils, further supporting that distinct immune cell infiltrates are predominantly attributed to ANCA GN. These important observations are of relevance and require further investigation with regard to distinct pathomechanisms contributing to inflammation and renal injury in ANCA GN, potentially generating knowledge on new therapeutic targets in specific ANCA subtypes.

The main limitations of our study are its retrospective design, the small patient number, and no data on efficacy of remission induction therapy or long-term renal outcomes. We here aimed to systematically analyze histological subtyping of immune cell infiltrates in ANCA GN and specifically chose univariate correlations of distinct immune cell infiltrates with clinico-pathological findings. However, multivariate comparisons and analysis of immune cell infiltrates adjacent to distinct histopathological lesions including previously described Bowman’s capsule rupture would also be of great interest and requires further investigation (20). Nevertheless, we here provide a novel systematic analysis of distinct subtypes of intrarenal immune cell infiltrates in association with clinical and histopathological findings, including glomerular and tubulointerstitial lesions in ANCA GN.

## Data Availability Statement

The original contributions presented in the study are included in the article/supplementary material. Further inquiries can be directed to the corresponding author.

## Ethics Statement

The studies involving human participants were reviewed and approved by the University Medical Center Göttingen, Germany. The patients/participants provided their written informed consent to participate in this study. Informed written consent was obtained from all subjects involved in the study for the use of routinely collected data for research purposes as part of their regular medical care in the contract of the University Medical Center Göttingen.

## Author Contributions

BT conceived the study, collected and analyzed data, and wrote the first draft. SH and DT collected and analyzed data. SH and PS evaluated histopathological findings. PK analyzed data and edited the manuscript. All authors contributed to the article and approved the submitted version.

## Funding

This research was funded by the Research program, University Medical Center, University of Göttingen, grant number 1403720. This research was also funded by the German Research Foundation, KFO (CRU) 5002, grant number STR 638/3-1 (DFG). We also acknowledge support from the Open Access Publication Funds of the Göttingen University.

## Conflict of Interest

The authors declare that the research was conducted in the absence of any commercial or financial relationships that could be construed as a potential conflict of interest.

## Publisher’s Note

All claims expressed in this article are solely those of the authors and do not necessarily represent those of their affiliated organizations, or those of the publisher, the editors and the reviewers. Any product that may be evaluated in this article, or claim that may be made by its manufacturer, is not guaranteed or endorsed by the publisher.
